# The age differences and effect of mild cognitive impairment on perceptual-motor and executive functions

**DOI:** 10.3389/fpsyg.2022.906898

**Published:** 2022-07-28

**Authors:** Yupaporn Rattanavichit, Nithinun Chaikeeree, Rumpa Boonsinsukh, Kasima Kitiyanant

**Affiliations:** Department of Physical Therapy, Faculty of Physical Therapy, Srinakharinwirot University, Nakhon Nayok, Thailand

**Keywords:** working memory, cognitive flexibility, cognitive inhibition, visual perception, visuoconstructional reasoning, perceptual-motor coordination

## Abstract

It is unclear whether the decline in executive function (EF) and perceptual-motor function (PMF) found in older adults with mild cognitive impairment (MCI) is the result of a normal aging process or due to MCI. This study aimed to determine age-related and MCI-related cognitive impairments of the EF and PMF. The EF and PMF were investigated across four groups of 240 participants, 60 in each group, including early adult, middle adult, older adult, and older adult with probable MCI. The EF, working memory, inhibition, and cognitive flexibility were evaluated using digit span backward tasks, the Stroop color-word test, and the modified switching verbal fluency test, respectively. The PMF, visual perception, visuoconstructional reasoning, and perceptual-motor coordination were evaluated using the clock reading test, stick design test, and stick catching test, respectively. Group differences were found for all subdomains of EF and PMF (*p* < 0.05), except for perceptual-motor coordination, indicating that this subdomain could be maintained in older adults and was not affected by MCI. For the age difference, working memory, cognitive flexibility, visual perception, and visuoconstructional reasoning remained stable across middle adults and started to decline in older adults, while cognitive inhibition began to decrease in middle adults and it further declined in older adults. To control the confounding effect of education level, the results showed that only cognitive flexibility was further decreased in older adults with probable MCI compared to those without MCI (*p* < 0.05). In conclusion, cognitive inhibition decreased earlier in middle adults, whereas EF and PMF started to decline in older adults. Cognitive flexibility was the only MCI-sensitive cognitive function.

## Introduction

Neurocognitive functions are essential for a person to work and perform activities of daily living independently. The decline of neurocognitive function varies among persons, ranging from a minor problem such as subjective memory complaints to a major problem such as dementia, which affects the ability to make an important decision, perform daily activities and live independently (Sachdev et al., 2014). Advanced age is an important factor leading to the decline of several neurocognitive functions, but the rate of cognitive decline and the extent of cognitive change vary among different neurocognitive domains (Hughes et al., 2018). Language and episodic memory generally remain intact until the age of 80 y, while processing speed declines with increasing age and rapidly declines after 60 y (Anstey and Low, 2004; Salthouse, 2010). A decline in cognitive processing speed was reported in middle adults when the speed of matching symbols with numbers (Zimprich and Mascherek, 2010) and counting numbers was used to assess this cognitive function (Ferreira et al., 2017). Executive function (EF) and perceptual-motor function (PMF) are the other components of cognitive function that involve the ability of a person to organize, manage, and execute activities in daily living (Sachdev et al., 2014). A decline in these functions results in impairment of instrumental activities of daily living (IADL) function among older adults (Jefferson et al., 2006).

The effect of increasing age on executive function has been extensively studied for 3 subdomains: cognitive inhibition, cognitive flexibility, and working memory. Cognitive inhibition is the ability to inhibit ongoing irrelevant information while executing actions to achieve a specific goal. Performance on cognitive inhibition has been reported to decline in older adults, as they had a lower ability to indicate the color of words while ignoring the meaning of the word compared to young adults (Scarpina and Tagini, 2017; Rey-Mermet and Gade, 2018; Gajewski et al., 2020). A previous study reported that older adults had a decline in performance on cognitive flexibility, as they had a lower number of correct word pairs switching between categories of words when compared to younger adults (De Paula et al., 2015). However, another study reported different results, as they showed that cognitive flexibility in older adults was not different from that in younger adults (Guarino et al., 2020). Working memory or the capacity to temporally store and manage information for carrying out complex cognitive tasks decline in older adults, as they could retrieve fewer correct numbers of backward digit spans compared to young adults (Choi et al., 2014; Faria et al., 2015). Although an age-related decline in subdomains of executive function was previously reported, the results were from multiple separate studies with insufficient data from middle adults. For direct comparison, a study that includes adults from different age groups using the same tests for assessing subdomains of the EF is necessary.

The effect of increasing age on perceptual-motor function (PMF) has also been reported, where the PMF declines with increasing age. PMF is categorized into visual perception, visuoconstructional reasoning, and perceptual-motor coordination (Sachdev et al., 2014). Visual perception is the ability to receive, interpret, and execute action according to visual stimuli. There are many types of visual perception, such as visuospatial perception, visual discrimination, and visual figure–ground perception. Previous studies showed impairment of visuospatial perception in older adults with dementia (Schmidtke and Olbrich, 2007) and Alzheimer’s disease (Mandal et al., 2012; Kim and Lee, 2021), as they had a reduced ability to translate visual stimuli, such as the positions of clock’s hands on 12 analog-clock faces into a concept of time compared to healthy adults (Schmidtke and Olbrich, 2007; Mandal et al., 2012; Kim and Lee, 2021). However, visuospatial perception among middle adults has not been reported elsewhere.

Visuoconstructional reasoning is the brain’s ability to organize and use spatial information to understand a structure. The decline of visuoconstructional reasoning was demonstrated in older adults without and with neurological deficits, as they have limited ability to draw a complex figure (Zhang et al., 2021) or to make an exact copy of a 2D wooden match arrangement (Baiyewu et al., 2005). Perceptual-motor coordination is the brain’s ability to interpret and use sensory information to perform physical activities. It is usually determined by measuring the reaction time of hand movements in response to visual stimuli such as light or moving objects. The perceptual-motor coordination function has been reported to decline in older adults when the stimuli involve light (Mapstone et al., 2003). However, the function in daily activity also requires the ability to move in response to a moving object, which is unknown in older adults.

Although an age-related decline in the subdomains of EF and PMF was previously reported, the results were from multiple separate studies with insufficient observations in middle adults. For direct comparison, a study that includes adults with different age groups using the same tests for assessing the subdomains of the EF and PMF is necessary.

An effect of neurocognitive deficit on the EF and PMF has been found in mild cognitive impairment (MCI), a state in which the cognitive function declines from a previous level of performance without affecting the independence on daily activities (Albert et al., 2011). MCI has been considered to be a transitional state between normal and dementia (Sachdev et al., 2014). Many studies have assessed EF performance in older adults with MCI to identify cognitive problems in MCI early to prevent further deficits. Working memory problems have been reported in older adults with MCI (Ahmed et al., 2016), as they had a lower number of backward digit retrieval when compared to older adults without MCI. A deficit of cognitive flexibility was found in older adults with MCI (Guarino et al., 2020). An impairment of cognitive inhibition has been reported in older adults with MCI compared to those without MCI (Rey-Mermet and Gade, 2018; Gajewski et al., 2020). In contrast, another study reported that cognitive inhibition was not impaired in older adults with MCI (Zhang et al., 2007). Thus, there are inconsistent results about the deficit of cognitive inhibition in this group of people.

Performance on the PMF has been measured in adults with cognitive impairment. A decline in visuoconstructional reasoning was found in older adults with MCI compared to those without MCI (Baiyewu et al., 2005; Salthouse, 2010). Visuospatial perception impairment has been found in patients with dementia (Schmidtke and Olbrich, 2007), but it has not been measured in older adults with MCI. Deficits in perceptual-motor coordination have been reported in adults with neurocognitive disease, such as adults with mild intellectual disability (Carmeli et al., 2008) and dementia (Liou et al., 2020). This subdomain of the PMF seems to be affected in adults with neurocognitive deficits; however, there is insufficient information to summarize the effect of MCI on the subdomains of the PMF.

Previous studies cannot clearly discriminate between the effect of age and MCI on each subdomain of PMF and EF. Therefore, this study aimed to determine which domains of the EF and PMF decline primarily as a result of age or as a result of MCI by comparing cognitive and perceptual-motor performance among young adults, middle adults, older adults, and older adults with probable MCI. We hypothesized that some EF and PMF domains would start to decline earlier since middle age and MCI would further trigger a decline of EF and PMF.

## Materials and methods

### Participants

The participants of this study were divided into 4 groups, including the early adult (20–39 years of age), middle adult (40–59 years of age), older adult (60 years of age or older) and older adult with probable mild cognitive impairment (MCI), both male and female living around Ongkharak district, Nakhon Nayok province, Thanyaburi district, Prathumtani Province and Bangkok and vicinity. The inclusion criteria were as follows: 1. age 20 years or older, ability to communicate, including speaking and reading in Thai, and no visual or hearing impairment; and 2. the Montreal Cognitive Assessment Thai version (MoCA-T) score was between 18–24 points for the probable MCI group and greater than or equal to 25 points for the other groups (Hoops et al., 2009; Tangwongchai et al., 2009; Ciesielska et al., 2016). The exclusion criteria were as follows: (1) participants who had depression (Thai Geriatric Depression Scale or TGDS-15 score over 4 points; Wongpakaran et al., 2013) and other psychological conditions; (2) instrumental activities of daily living Thai version score (IADL) over 20 points (Senanarong et al., 2013); (3) history of movement disorders such as stroke and Parkinson’s disease; (4) histologically confirmed diagnosis of unstable diabetes mellitus; (5) alcohol use disorders identification test score over 20 points; (6) the number of packs of cigarettes smoked over 10 pack years.

### Sample size calculation

In this cross-sectional study, the sample size was calculated with the G power software using the ANOVA procedure (Kim, 2016), based on the results of a similar previous study (Viratchakul et al., 1970). Mean neuropsychological score of probable MCI group (*x̄* = 3.04, *N* = 77) and healthy control (*x̄* = 3.59, *N* = 103), α (0.05), and power (0.95) were used for the sample size calculation, resulting in the total sample size of 236. To round this up, the required total number was 240 people, 60 in each group.

### Research methodology

#### Neuropsychological testing

Six neuropsychological tests were administered to all participants to investigate the EF and PMF domains. The EF domains were assessed using the digit span test (DST), the Stroop color-word test (SCWT), and the modified switching verbal fluency test (mSVF). The PMF domains were assessed using the clock reading test (CRT), the stick design test (SDT), and the stick catching test (SCT). Six raters participated in this study, each of whom was assigned to assess one neuropsychological test. The intrarater reliability of all raters was good, with an ICC (3,1) of 0.745 to 0.997.

1. The Stroop color-word test (SCWT) was used in the inhibitory control test (Scarpina and Tagini, 2017). This test has three subtasks. Participants are asked to say the name of the color of a visible swatch (C), report the color of the characters printed in a color corresponding to the word (W), and report the color of the text, which did not match the meaning of the printed word (CW). Participants were asked to say the correct name of the color and the color for each item included in each subtask as much as possible. The time limit for each subtask was set at 45 s. The number of correct items in each subtask was recorded and used to calculate the interference score (IG) with the following formula: IG = CW − [(W × C)/(W + C)]. A negative IG value represents a pathological ability to inhibit interference, where a lower score means a greater difficulty in inhibiting interference.
2. The digit span test (DST) is a test used to evaluate attention and working memory, which consists of backward and forward digit recall sequences (Zucchella et al., 2018). Although the specific cognitive components contributing to each part of the DST performance have not been well defined, digit span forward is believed to be more related to attention, and digit span backward is believed to be more related to working memory (Choi et al., 2014). In this test, participants were asked to say a series of numbers at a speed of 1 number per second out loud and then repeat the set of numbers in forward (DSF) and backward (DSB) conditions. For example, the participants listened to the numerical set “6, 9, 4, 7” and then were asked to repeat the number set. It was “6, 9, 4, 7” for forward, or “7, 4, 9, 6” for backward. The length of the numeric set ranged from 2–10 characters. A series of tests were performed, 3 times each. If the test participants correctly answered at least 2 times, they were considered to pass that set of numbers. On the other hand, if the participants could not answer correctly at least 2 times, the test was stopped.
3. The modified switching verbal fluency test (mSVF) is a cognitive flexibility test (De Paula et al., 2015). In this test, the participants must present words in two categories, including fruit and animal categories, alternately as many as possible within a 1 min period. The researcher recorded the correct word count according to the category.
4. The stick design test (SDT) is a test for visuoconstructional reasoning (Baiyewu et al., 2005). Prior to data collection, a researcher demonstrated how to arrange matches into four specified formats, including a circle, a diamond, overlapping rectangles and a cube. The participants were presented with a series of matches according to the sample demonstrated. The total scoring was 12 points based on the correctness of the images obtained from the arrangement of matches in various aspects, namely, the shape of the sample, aligned correctly in the specified direction, and the correct match head position.
5. The clock reading test (CRT) was a visual perception test in which the participants were asked to read the time shown on the watch face, which was a picture of a clock without numbers, for 12 different times over 5 min (Schmidtke and Olbrich, 2007). If the participants could tell the time accurately within less than 3 min, 1 point was awarded.
6. The stick catching test (SCT) is a perceptual-motor coordination test (Kauranen and Vanharanta, 1996). Before the start of the test, participants were asked to sit in the starting position: sitting on a chair, placing their dominant arm on a table. The shoulder joint was at an angle of 30 degrees bent with 20 degrees of extension. The elbow was bent 90 degrees. The forearm was positioned halfway between the prone and supine position. The wrist was in line with the forearm and was extended forward. The participants were asked to grasp the stick with the tip of the index finger and thumb as quickly as possible, immediately after the investigator released the stick, with the elbow and wrist still in the same position as the starting position. The researcher recorded the distance between the start and the last point the subjects captured the stick, and the mean obtained from each arm test was used to calculate the visual reaction time using the formula as follows: Visual reaction time.

All participants were asked to take all neuropsychological tests. The researchers provided a description and example of the test to ensure that the participants understood the protocol of each test. During the testing, the participants were allowed to rest for at least two minutes between tests or until they were satisfied before the next test was started to avoid cognitive fatigue and motivational effects (Ruiz et al., 2020).

### Statistical analysis

Subject characteristics such as age, MoCA-T and education years were expressed as the mean ± SD and compared between groups using the Kruskal–Wallis test and multiple comparisons (Mann–Whitney *U* test). Analysis of variance and Kruskal–Wallis tests were used to compare among groups regarding the domains of the EF and the PMF, respectively. The Bonferroni test and Mann–Whitney *U* test were used to define specific differences between groups.

Educational background is one of the important interindividual variabilities in cognitive performance (Hughes et al., 2018). In order to control the effect of educational level, one-way analysis of covariance (ANCOVA) was performed on mSVF, DSF, CRT, and SDT scores to determine whether MCI would trigger a further decline in EF and PMF. ANCOVA assumption of normality and equal variance was checked, thus, ANCOVA with *post hoc* Bonferroni test was performed on mSVF and DSF scores but nonparametric ANCOVA (Quade’s method) with *post hoc* analysis using Bonferroni test was selected for comparing CRT and SDT scores.

## Results

Three hundred and sixty-one people were recruited. Those excluded were 12 early adults and 30 middle adults with MoCA-T scores <25 points, and 81 older adults with MoCA-T scores of less than 18 points, resulting in 240 participants in this study. They were early adults (*n* = 60), middle adults (*n* = 60), older adults (*n* = 60), and older adults with probable MCI (*n* = 60). Participant characteristics, including age, years of education, sex, MoCA-T score, underlying disease, depression, and IADL results, are presented in Table 1. There was no significant difference in age between older adults and older adults with probable MCI, but years of education were significantly lower in older adults with probable MCI than in the other groups. MoCA-T scores were significantly different among subject groups such that early adult demonstrated highest MoCA-T scores, while the lowest MoCA-T scores were found in older adults with probable MCI. All participants had no depression and were independent in activities of daily living (Table 1).

**Table 1:** Participants’ characteristics.

**Characteristics**	**Groups**			
	**Early adult (*n* = 60)**	**Middle adult (*n* = 60)**	**Older adult (*n* = 60)**	**Older with probable MCI (*n* = 60)**
**Age (years)**				
Mean ± SD	29.4 ± 5.7	49.4 ± 6^a^	68.8 ± 6.4^a^^,^^b^	69.3 ± 7.0^a^^,^^b^
(Min–Max)	(20–39)	(40–59)	(60–85)	(60–88)
**Education (years)**				
Mean ± SD	15.3 ± 2.0	14.4 ± 3.8	14.0 ± 4.6	7.7 ± 4.1^a^^,^^b^^,^^c^
(Min–Max)	(9–18)	(4–18)	(4–21)	(4–16)
**Sex**, male/female (*n*)	14/46	15/45	24/36	22/38
**MoCA-T (points/30)**				
Mean ± SD	27.6 ± 1.8	26.8 ± 1.5^a^	26.0 ± 1.4^a^^,^^b^	20.5 ± 2.0^a^^,^^b^^,^^c^
(Min–Max)	(25–30)	(25–30)	(25–30)	(18–24)
**Underlying diseases (n)**				
No diseases or not detect	58	43	20	25
NCD	2	16	38	35
Musculoskeletal diseases	0	1	2	2
**Depression (n)**				
No	60	60	60	60
Yes	0	0	0	0
**IADL (n)**				
Independent	60	60	60	60
Dependent	0	0	0	0

Data were collected from 60 participants in each group. Age, years of education, and score on the Montreal cognitive assessment – Thai version (MoCA-T) of participants in each group are represented as the mean ± standard deviation and range (minimum-maximum). Noncommunicable diseases (NDC) include hypertension, heart disease, dyslipidemia, diabetes mellitus, and asthma. Musculoskeletal diseases include gout and rheumatoid. IADL; Instrumental Activities of Daily Living. Age, years of education and score on the MoCA-T were analyzed using Kruskal–Wallis ANOVA, and multiple comparisons (Mann–Whitney U test) showed significant differences among groups.

a *p* < 0.05 compared with the early adult group.

b *p* < 0.05 compared with the middle adult group.

c *p* < 0.05 compared with the older adult group.

### Effect of age and MCI on domains of executive function

**Figure 1:**
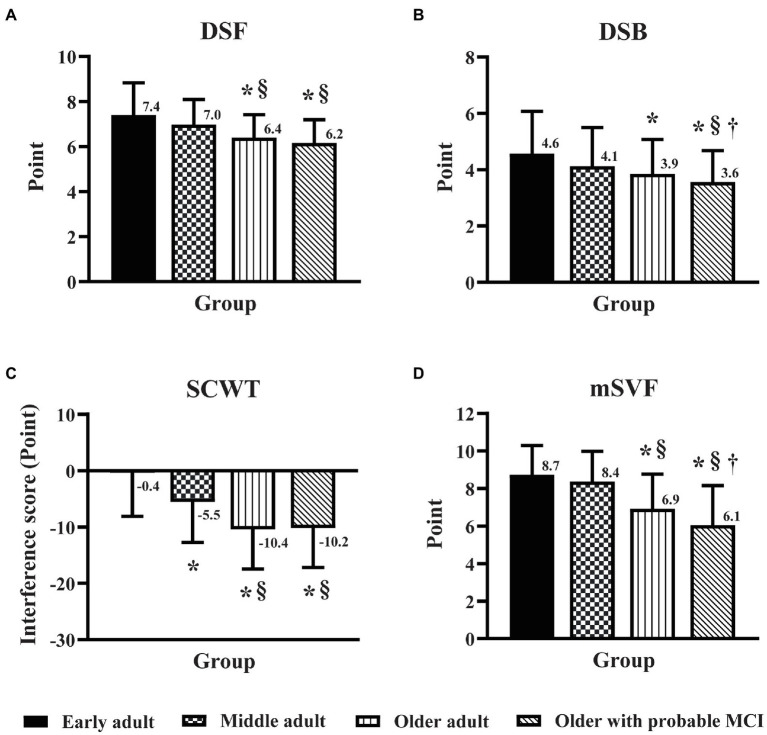
Effect of age and mild cognitive impairment on executive function domains. Executive function was assessed by the digit span forward task **(A)**, the digit span backward task **(B)**, the Stroop color-word test **(C)**, and the modified switching verbal fluency test **(D)** in early adult, middle adult, older adult, and older MCI. DSF, digit span forward task; DSB, digit span backward task; SCWT, Stroop color-word test; mSVF, modified switching verbal fluency test; MCI, mild cognitive impairment. ^*^p < 0.05 vs. early adult group; ^§^*p* < 0.05 vs. middle adult group; ^†^*p* < 0.05 vs. older adult group.

The DSF and DSB scores of older adults and older adults with probable MCI were lower (*p* < 0.05) than those of early adults and middle adults (Figures 1A,B). While there was no significant difference in the DSF score between older adults and older adults with MCI (Figure 1A), the DSB score in older adults with probable MCI was lower (*p* < 0.05) than that in older adults (Figure 1B). The interference score (IG) of the SCWT in middle adults, older adults, and older adults with probable MCI was lower (*p* < 0.05) than that in early adults (Figure 1C). While no significant difference in the IG was observed between older adults and older adults with probable MCI, both groups showed lower IG scores than middle adults (Figure 1C). The mSVF score was significantly lower (*p* < 0.05) in older adults and older adults with probable MCI than in the other groups (Figure 1D), and this score in older adults with probable MCI was lower (*p* < 0.05) than that in older adults (Figure 1D). The results of the EF domain suggested that older age affected attention, working memory and cognitive flexibility, and MCI triggered further declines in working memory and cognitive flexibility. In addition, the increased age, starting from middle age, led to difficulty in inhibiting interference and reduced the performance on cognitive inhibition.

### Effect of age and MCI on domains of perceptual-motor function

Table 2 shows the effect of age and MCI on perceptual-motor domains. The CRT and SDT scores were lower (*p* < 0.05) for older adults and older adults with MCI than early adults and the older adults with probable MCI demonstrated the lowest CRT and SDT scores (*p* < 0.05). In contrast, there were no significant differences in reaction time for the SCT score with dominant and nondominant hands among all groups. These data showed that the increased age resulted in reduced visual perception and visuoconstructional reasoning with no effect on perceptual-motor coordination, and MCI aggravated further decline of these visual perception and visuoconstructional reasoning domains.

**Table 2:** The effect of age and MCI on the domains of perceptual-motor function.

**Cognitive performance**	**Groups**			
	**Early adult (*n* = 60)**	**Middle adult (*n* = 60)**	**Older adult (*n* = 60)**	**Older with probable MCI (*n* = 60)**
**CRT (points)**				
Mean ± SD	10.8 ± 1.1	10.8 ± 1.4	10.4 ± 2.4^a^	9.7 ± 2.4^a^^,^^b^^,^^c^
(Min – Max)	(6.5–12)	(5–12)	(6.5–12)	(0–12)
**SDT (points)**				
Mean ± SD	11.7 ± 0.9	11.6 ± 0.8	11.0 ± 1.0^a^^,^^b^	10.5 ± 1.2^a^^,^^b^^,^^c^
(Min – Max)	(7–12)	(9–12)	(8–12)	(7–12)
**SCT**				
**- Dominant hand (seconds)**				
Mean ± SD	0.2 ± 0.0	0.2 ± 0.0	0.2 ± 0.0	0.2 ± 0.0
(Min–Max)	(0.1–0.3)	(0.1–0.3)	(0.1–0.3)	(0.1–0.3)
**- Non-dominant hand (seconds)**			
Mean ± SD	0.2 ± 0.0	0.2 ± 0.0	0.2 ± 0.0	0.2 ± 0.0
(Min–Max)	(0.1–0.3)	(0.1–0.3)	(0.1–0.3)	(0.1–0.3)

Older adult, older adult with normal cognitive function; older MCI, older adult with probable mild cognitive impairment; CRT, clock reading test; SDT, stick design test; SCT, stick catching test. Data were analyzed using Kruskal–Wallis ANOVA, and multiple comparisons (Mann–Whitney U test) showed significant differences among groups.

a *p* < 0.05 compared with the early adult group.

b *p* < 0.05 compared with the middle adult group.

c *p* < 0.05 compared with the older adult group.

### Influence of education level on the effect of age and MCI on domains of EF and PMF

**Figure 2:**
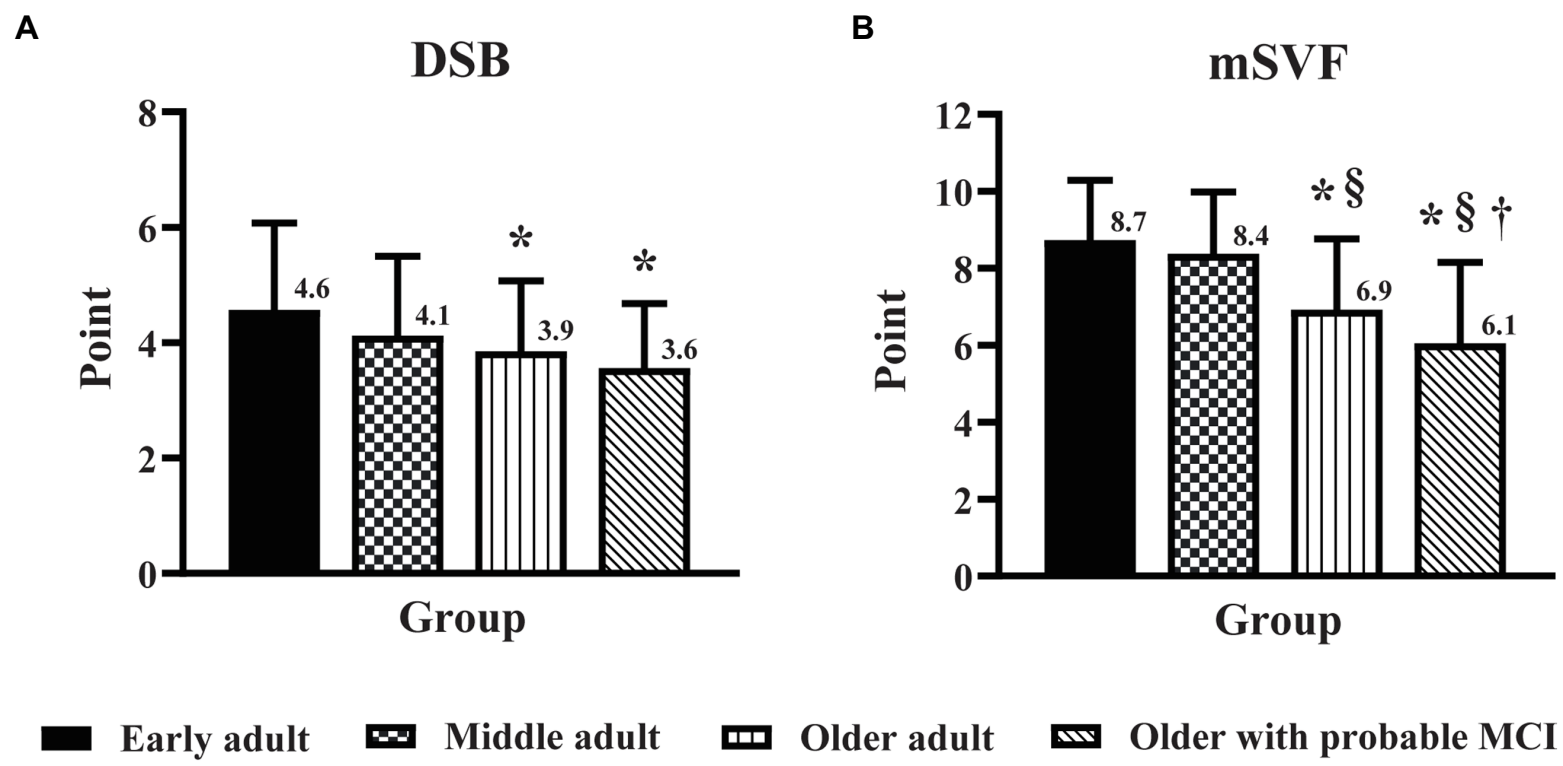
The influence of education level on the effect of age and mild cognitive impairment (MCI) on the domains of executive function. Executive function was assessed by the digit span backward task **(A)** and the modified switching verbal fluency test **(B)** in early adult, middle adult, older adult, and older with probable MCI patients with a high education level. DSB, digit span backward task; mSVF, modified switching verbal fluency test; MCI, mild cognitive impairment. ^*^p < 0.05 vs. early adult group; ^§^*p* < 0.05 vs. middle adult group; ^†^*p* < 0.05 vs. older adult group.

**Figure 3:**
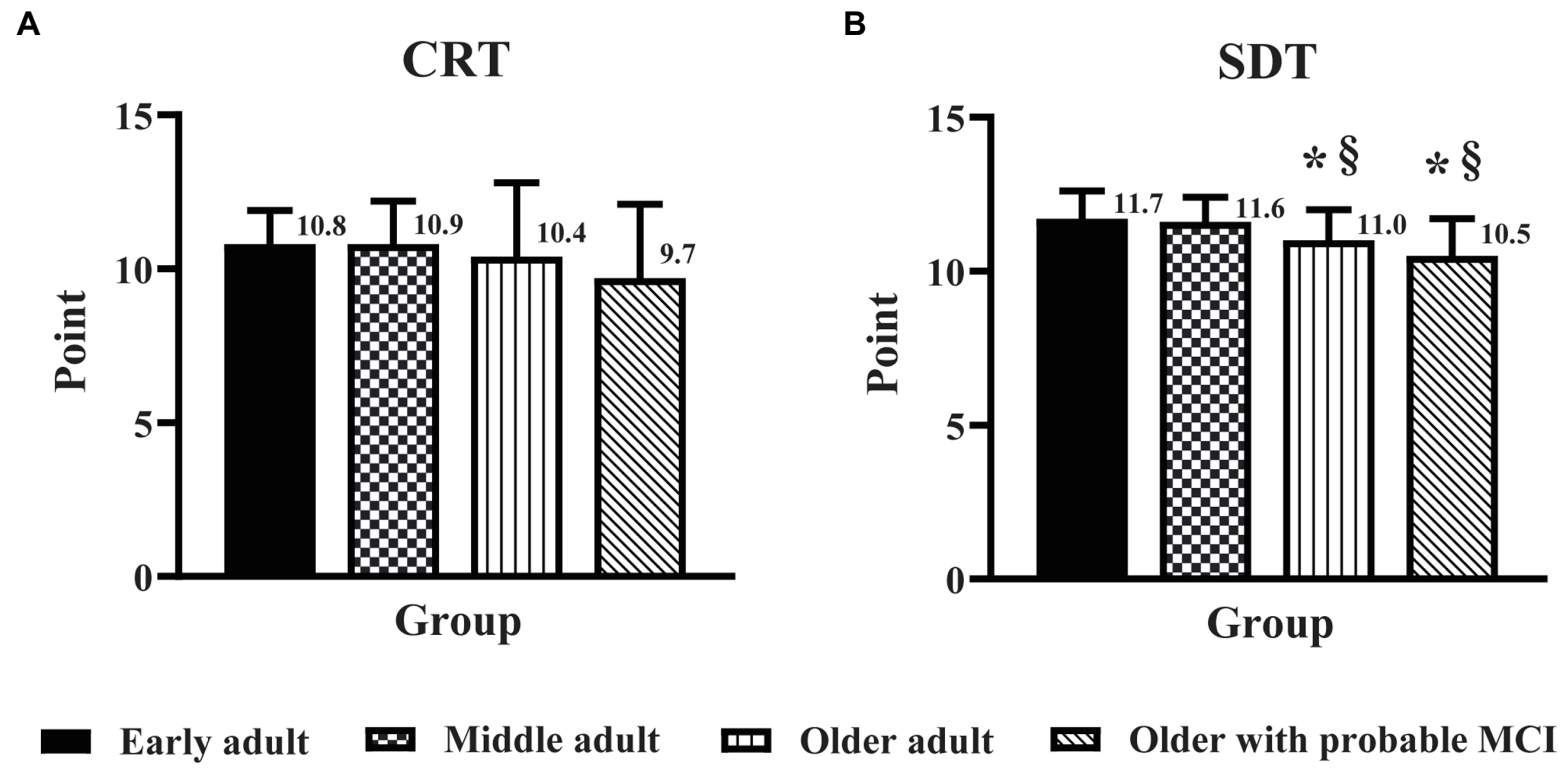
The influence of education level on the effect of age and mild cognitive impairment on the domains of perceptual-motor function. Perceptual-motor function was assessed by the clock reading test **(A)** and the stick design test **(B)** in early adult, middle adult, older adult, and older adults with probable MCI and a high education level. CRT, clock reading test; SDT, stick design test; MCI, mild cognitive impairment. ^*^p < 0.05 vs. early adult group; ^§^*p* < 0.05 vs. middle adult group.

The DSB score in older adults with probable MCI was lower (*p* < 0.05) than that in middle adults (Figure 2), but there was no significant difference in the DSB score between older adults and older adults with probable MCI. In contrast, MCI triggered a decline in cognitive flexibility, as seen from the finding that the mSVF score in older adults with probable MCI was lower (*p* < 0.05) than that in older adults (Figure 2). For the PMF domain, no significant difference was found for the CRT and SDT scores between older adults and older adults with probable MCI (Figure 3).

## Discussion

This study expanded the knowledge on the decline of executive function (EF) in aging MCI. We aimed to clarify whether the decline in EF in older adults with MCI was the result of a normal aging process or due to MCI pathology. The new findings from this study were that the decline in cognitive flexibility (EF subdomain) in older adults with MCI is due to MCI pathology, while the decline of other EF subdomain in MCI, i.e., inhibition and working memory, were the result of a normal aging process.

### Age differences on the EF and PMF

This study investigated the performance of executive (EF) and perceptual-motor functions (PMF) across various adult groups (early adult, middle adult, older adult) and older adults with mild cognitive impairment (MCI). Our findings suggested that the performance on the EF and PMF domains such as attention, working memory, cognitive flexibility, cognitive inhibition, visual perception, and visuoconstructional reasoning decreased with age, while perceptual-motor coordination did not change by age.

For the EF domains, we found that the performance of attention, working memory, and cognitive flexibility remained stable across middle adults and then started to decrease in older adults. These findings are consistent with a previous study indicating that attention and working memory performance decrease after the age of 65 years (Grégoire and Van der Linden, 1997). Our result is also in line with a previous study that evaluated cognitive flexibility by using animal fluency (Gunstad et al., 2006) and with a recent study using number and letter task switching paradigm that clearly reported MCI deficit in cognitive flexibility (Velichkovsky et al., 2020). Specifically, we found that the performance of cognitive inhibition began to decrease in middle adults and then decreased more in older adults, suggesting that cognitive inhibition is the earliest age-sensitive cognitive decline. These findings are consistent with the theoretical framework of Hasher & Zacks indicated that a decline of cognitive function in elderly was a consequence of insufficient in cognitive inhibition (Hasher and Zacks, 1988).

For the PMF domains, we demonstrated that the performance of visual perception and visuoconstructional reasoning declined in older adults. A decrease in visuoconstructional reasoning ability in older adults was similar to the study from Mapstone and colleagues (Mapstone et al., 2003), but visual perception decline was slightly different from a previous study in which it was found to decrease in adults aged 50 (Kim et al., 2014). This could be because the previous study did not standardize the educational level which may affect the performance of visual perception (Herrera-Guzmán et al., 2004). Regarding perceptual-motor coordination, we found that performance did not change across age. This finding is inconsistent with a previous study indicating that simple reaction time slowed with age (Godefroy et al., 2010). It is possible that the previous study used more sensitive equipment for measuring the reaction time, while our study used a method that can be easily applied in the community setting. Thus, the method of measurement selected in this study may not be sensitive enough to differentiate the performance of each age group on perceptual-motor coordination.

### Influence of MCI on the EF and PMF

This study demonstrated that MCI triggered a further decline in the EF and PMF domains of working memory, cognitive flexibility, visual perception, and visuoconstructional reasoning, in addition to the age factor. These findings are consistent with previous studies that showed a deficit of cognitive flexibility, which was evaluated by the Trail making task Part B (Zhang et al., 2007; Kim and Lee, 2021) and the Modified Card Sorting Test (Traykov et al., 2007) even in the earliest stage of the MCI. Other studies also found that impairments in working memory have been shown during aging (McNab et al., 2015) and progression of MCI (Garcia-Alvarez et al., 2019), which may be evident in both nonamnestic and amnestic types of MCI (Albert et al., 2007). In line with these studies, our results indicated that a decline in working memory and cognitive flexibility during aging was aggravated by MCI. The explanation for the decline in cognitive flexibility could be the increased memory retrieval processing and working memory required during the mSVF task, which usually degenerates during aging and worsens in MCI (Henry and Crawford, 2004).

Moreover, our results using ANCOVA to control the confounding effect of education level showed that only cognitive flexibility was sensitive to MCI. A recent conceptualization indicated that cognitive flexibility controlled by the frontoparietal network (Marek and Dosenbach, 2018) was considered a higher-order control process to ensure that cognitive function was appropriate to changes in the environment (Hommel, 2015). A previous study showed that one of the early signs of dementia is cognitive rigidity, which is associated with dysfunction of the frontoparietal network (Uddin, 2021), a flexible hub for cognitive control (Marek and Dosenbach, 2018). The activity of frontoparietal network has been shown to be reduced in early MCI, while other brain networks are altered later (Babiloni et al., 2006; Wang et al., 2019). This finding is consistent with our study that showed a selective decline in cognitive flexibility in MCI.

Our results also demonstrated that MCI did not affect the performance of visual perception and visuoconstructional reasoning after educational level was controlled. These findings were in line with results from other previous literature which administered an hour hand task and a minute hand task to trigger more mental imaging (Leyhe et al., 2009) or the three-dimensional constructional praxis test (Rapp et al., 2010). Both previous studies indicated that normal healthy aging and MCI did not show a significant difference in brain activation. On the other hand, when using the constructional praxis task, there was a decline of visuoconstructional reasoning and visual spatial perception in MCI, while impairment was not detected in normal aging (Saur et al., 2010; Kume et al., 2019). This inconsistency could be due to differences in measurement method, subject recruitment criteria, or clinical manifestations of persons with MCI (De Jager, 2004).

### Clinical implications and study limitations

Our research findings can be implemented for all areas where healthcare professionals assist the elderly. This study provided evidence to support the use of simple neuropsychological testing to differentiate cognitive performance among older adults without and with MCI. These tests are reliable, valid and easy to administer in the community setting (Baiyewu et al., 2005; Schmidtke and Olbrich, 2007; Limpawattana and Manjavong, 2021). Furthermore, our results demonstrated an early decline in cognitive inhibition; thus, we suggest that screening and prevention should begin in early adults to prevent or slow the decline in cognitive inhibition in older adults and its progression to MCI.

Our study has some limitations. Individuals with amnestic MCI have predominant memory loss and are at high risk of progressing to Alzheimer’s disease (Grundman et al., 2004), whereas individuals with nonamnestic MCI have problems in cognitive domains other than memory (Petersen, 2004). Therefore, future studies should examine the differences in each type of MCI in cognitive abilities, as different types of MCI may influence the decline of EF and PMF differently. Second, the older adult group in this study had a mean age of 70 years, so generalization outside of this age group is limited. Third, in addition to the level of education, other environmental factors, such as occupation, usage of other languages, engaging in cognitively stimulating activities, and physical activity, affect cognitive performance *via* improving the cognitive reserve (Baldivia et al., 2008; Cheng, 2016; Stern et al., 2018). Thus, these factors should be investigated in future research to determine their effects on the domains of executive and perceptual-motor function. Lastly, MoCA-T score was used in this study as a clinical tool to identify individuals with MCI (Chen et al., 2021). Clinicians should be cautious on the accuracy of MCI diagnosis using MoCA which could be improved using other biomarkers such as β-amyloid 42 and t-tau protein. In conclusion, our results suggest that the performance of the executive and perceptual-motor function domains selectively change with aging. Cognitive flexibility, working memory, visual perception and visuoconstructional reasoning remained stable in middle adults and declined in advanced age, whereas cognitive inhibition initially decreased in middle adults and progressed in older adults. Moreover, when the education factor was controlled, MCI selectively triggered a further decline in cognitive flexibility only.

## Data availability statement

The original contributions presented in the study are included in the article/supplementary material, further inquiries can be directed to the corresponding author.

## Ethics statement

The studies involving human participants were reviewed and approved by the Ethics Review Committee on Human Research, Srinakharinwirot University (Code SWUEC 300/2562 and 301/2562). The patients/participants provided their written informed consent to participate in this study.

## Author contributions

RB contributed to conception of the study and gave the approval to the final version of the manuscript. KK, YR, and NC performed data collection and analysis and contributed to manuscript drafting and revision. All authors contributed to the article and approved the submitted version.

## Funding

This work was supported by Faculty of Physical Therapy, Srinakharinwirot University (Grant Number 275/2563 and 411/2563).

## Conflict of interest

The authors declare that the research was conducted in the absence of any commercial or financial relationships that could be construed as a potential conflict of interest.

## Publisher’s note

All claims expressed in this article are solely those of the authors and do not necessarily represent those of their affiliated organizations, or those of the publisher, the editors and the reviewers. Any product that may be evaluated in this article, or claim that may be made by its manufacturer, is not guaranteed or endorsed by the publisher.
